# Avian BMR in Marine and Non-Marine Habitats: A Test Using Shorebirds

**DOI:** 10.1371/journal.pone.0042206

**Published:** 2012-07-31

**Authors:** Jorge S. Gutiérrez, José M. Abad-Gómez, Juan M. Sánchez-Guzmán, Juan G. Navedo, José A. Masero

**Affiliations:** Department of Anatomy, Cell Biology and Zoology, University of Extremadura, Badajoz, Spain; University of Roehampton, United Kingdom

## Abstract

Basal metabolic rate (BMR) is closely linked to different habitats and way of life. In birds, some studies have noted that BMR is higher in marine species compared to those inhabiting terrestrial habitats. However, the extent of such metabolic dichotomy and its underlying mechanisms are largely unknown. Migratory shorebirds (Charadriiformes) offer a particularly interesting opportunity for testing this marine–non-marine difference as they are typically divided into two broad categories in terms of their habitat occupancy outside the breeding season: ‘coastal’ and ‘inland’ shorebirds. Here, we measured BMR for 12 species of migratory shorebirds wintering in temperate inland habitats and collected additional BMR values from the literature for coastal and inland shorebirds along their migratory route to make inter- and intraspecific comparisons. We also measured the BMR of inland and coastal dunlins *Calidris alpina* wintering at a similar latitude to facilitate a more direct intraspecific comparison. Our interspecific analyses showed that BMR was significantly lower in inland shorebirds than in coastal shorebirds after the effects of potentially confounding climatic (latitude, temperature, solar radiation, wind conditions) and organismal (body mass, migratory status, phylogeny) factors were accounted for. This indicates that part of the variation in basal metabolism might be attributed to genotypic divergence. Intraspecific comparisons showed that the mass-specific BMR of dunlins wintering in inland freshwater habitats was 15% lower than in coastal saline habitats, suggesting that phenotypic plasticity also plays an important role in generating these metabolic differences. We propose that the absence of tidally-induced food restrictions, low salinity, and less windy microclimates associated with inland freshwater habitats may reduce the levels of energy expenditure, and hence BMR. Further research including common-garden experiments that eliminate phenotypic plasticity as a source of phenotypic variation is needed to determine to what extent these general patterns are attributable to genotypic adaptation.

## Introduction

Measurements of metabolic rates provide valuable information on the physiological performance of an organism in a particular environment and offer a universal metric for comparisons across and within taxa [1]–[3]. Basal metabolic rate (BMR) –the minimum rate of energy expenditure of normothermic homeotherms under thermoneutral and postabsorptive conditions in the inactive phase of the circadian cycle [4], [5]– is the energetic trait most widely studied by ecological and comparative physiologists [6]–[8]. Its importance lies in both its significant contribution to daily energy turnover (up to 63%; [3]) and its correlation with a range of organismal (diet, life stage, behaviour, migratory tendency, phylogeny) and environmental factors (latitude, climate, habitat, season, temperature, rainfall, ecosystem productivity) after differences in body mass have been controlled statistically (see [9], [10] for recent reviews). Among these factors, habitat has a marked influence on BMR, as it integrates a suite of abiotic and biotic characteristics that will interact with the organism itself to ultimately affect its energetics [9]–[12].

In birds, there is considerable evidence that BMR is closely linked to habitat characteristics [10]–[12], indicating that variations in BMR may reflect the changing selection pressures in different habitats. Indeed, inter- and/or intraspecific comparative studies of avian BMR have revealed various dichotomies in relation to habitat type: arid–mesic [13]–[16]; tropical–temperate [7], [17], [18]; or marine–non-marine [10], [19]–[22]. The latter case, albeit less well recognized, was first noted more than 25 years ago by Ellis [19] and Rahn & Whittow [20], who found that the BMR of many seabirds (orders Sphenisciformes, Procellariiformes, Pelecaniformes, and Charadriiformes) was higher than predicted from the equations for non-passerines available at the time [23], [24]. In his comprehensive review of seabird energetics, Ellis [19] also demonstrated a latitudinal gradient for BMR in Charadriiforms, as established in mainly terrestrial birds [7], [17], [25], [26]. These findings were later supported by other studies in sea- and shorebirds (e.g. [21], [27]–[31]; see also [10]), which collectively suggest that the relatively high BMRs of these mainly north temperate/arctic breeders reflect the up-regulation of metabolic machinery required for enhanced cold tolerance and long-distance migrations. To date, however, evidence for this metabolic divergence (marine–non-marine) mostly comes from allometric equations that do not integrate environmental factors and/or corrections for phylogenetic relatedness that potentially affect BMR, thereby weakening the accuracy of obtained results and potentially leading to misleading conclusions [32], [33].

Migratory shorebirds (Charadriiformes) offer a particularly interesting opportunity for testing this marine–non-marine split, as they occur in both coastal and inland habitats. Overall, high Arctic-breeding species rely primarily on marine saline (intertidal) habitats during the non-breeding season (usually referred to as ‘coastal’ shorebirds), while more southerly-breeding species tend to rely on inland freshwater (non-tidal) habitats (usually referred to as ‘inland’ shorebirds), irrespective of diet and foraging style [34]–[36]. Such contrasting strategies may have important energetic consequences, as coastal and inland shorebirds will experience different environmental and ecological conditions whatever the latitude (e.g. differences in ambient temperature, wind exposure, salinity, parasite abundance, foraging patterns, or food availability) [37], [38]. In this context, BMR may reflect the overall pace of life of species (or populations) that inhabit such contrasting habitats (e.g. [7], [18]). Despite the BMR of coastal shorebirds having been repeatedly studied along the migration route [27]–[30], [39], surprisingly little effort has been directed toward measurement of BMR of shorebirds inhabiting inland habitats (but see [40]–[44]). Interestingly, these latter studies show some evidence that inland shorebirds may have lower BMRs than their coastal counterparts. Such differences in levels of metabolism between coastal and inland shorebirds could be attributable to genotypic adaptation, to phenotypic plasticity, or to some interaction between these mechanisms.

In the present study, we measured BMR for 12 species of migratory shorebirds wintering in temperate inland freshwater habitats and collected additional BMR values from the literature for coastal and inland shorebirds along their migratory route. The objectives of the study were threefold. First, to provide new data on the BMR of shorebird species living in inland habitats, three of which have not previously been studied in wild-caught individuals (stone curlew *Burhinus oedicnemus*, black-tailed godwit *Limosa limosa*, and spotted redshank *Tringa erythropus*). Our second and most important aim was to test for differences in shorebirds’ BMR between marine and non-marine habitats. According to the above-mentioned studies, we predicted that shorebirds wintering in inland freshwater habitats would have lower BMRs than those wintering in coastal saline habitats after controlling for the effects of potentially confounding climatic (latitude, temperature, solar radiation, wind conditions) and organismal (body mass, migratory status, phylogeny) factors. Our final objective was to discuss some mechanisms which could account for inter- and intraspecific variability of BMR in shorebirds wintering in such contrasting habitats.

## Materials and Methods

### Ethics Statement

Birds were captured under permits from Gobierno de Extremadura (CN09/02194; CN10/0754; CNO103/11/OT) and Junta de Andalucía (SGYB/FOA/AFR/AV). After metabolic measurements were conducted, we returned the birds to continual food access so that they might build up body reserves. Thereafter, we released the birds from whence they were originally captured. All protocols described in this article were approved by the Committee of Bioethics and Biosecurity of the University of Extremadura (63/72011).

### Animals

One hundred and eighteen individuals belonging to 12 species of migratory shorebirds were captured (January–March, 2009–2011) with mist nets and clap nets on temperate inland freshwater habitats in Extremadura (39°01′N, 5°58′W; Appendix S1), a key area of Southern Europe for many non-breeding waterbirds (e.g. [45], [46]). Ten overwintering dunlins *Calidris alpina* were also captured in mid-March 2010 at a saline marsh in Cádiz Bay Natural Park, S Spain (36°23′N, 6°8′W; Appendix S1). After capture, each bird was fitted with an individual steel ring and then transported to our bird facilities at the University of Extremadura (38°52′N, 7°00′W; see [43], [44] for details), where BMR was measured within 48 h of capture (see below). The experimental protocol used for determining BMR essentially resembles that previously described by Kvist and Lindström [30] and Lindström and Klaassen [39]. Briefly, prior to BMR measurements, birds were weighed (to the nearest 0.1 g) and scored for the extent of their subcutaneous fat stores using a semiquantitative scale for shorebirds (0–7, with 7 being the fattest), as described by Meissner [47]. As most individuals had small fat scores (≤4), BMR was usually measured on the day of capture after a fasting period of at least 5 h. This ensured that they were in a postabsorptive digestive condition, giving priority to small-sized species and individuals with smaller fat stores. Birds with higher fat scores (4–7) were held in outdoor aviaries with a limited amount of alive food (fly larvae, *Protophormia* sp.) and a constant supply of continuously flowing freshwater for drinking and bathing. Once they reached a fat score ≤4, we proceeded to measure BMR.

### BMR Measurements

We measured rates of oxygen consumption using standard flow-through respirometry (see [43], [44]). Birds were measured under postabsorptive digestive conditions during the rest phase of their circadian cycle [48], i.e. overnight in all species with the exception of the stone curlew and the common snipe *Gallinago gallinago*. Both species exhibit considerable crepuscular/nocturnal activity throughout the year [49], [50], so we measured oxygen consumption during the daylight period, i.e. when the individuals were resting (see e.g. [41], [42] for a similar procedure). The metabolic chambers (3.6–15 L for birds weighing 20–560 g, or 60 L for a single Eurasian curlew *Numenius arquata* weighing 875 g) were in complete darkness and located in a temperature-controlled room at a constant temperature of 27°C (±0.5°C), i.e. within the thermoneutral zone of all the measured species [51] and similar to that used in previous studies on shorebirds [27]–[30], [39]. The metabolic chambers received atmospheric air at a rate of 60–l50 L h^−1^ (standard temperature and pressure) so that oxygen consumption did not exceed 0.5% of the incoming oxygen volume. Calibrated mass flow controllers (MFS-5, Sable Systems, Las Vegas, NV, USA) allowed us to measure the air flow upstream. Water vapour was removed from the air stream immediately downstream from the metabolic chambers using Drierite columns (anhydrous calcium sulphate, W. A. Hammond Drierite, Xenia, OH, USA) followed by a multiplexer (TR-RM4, Sable Systems, Las Vegas, NV, USA), which allowed automatic switching between up to four channels. Oxygen concentration was determined using a gas analyzer (FC-10 Oxygen Analyzer, Sable Systems, Las Vegas, NV, USA). Dry outside air (set to 20.95% oxygen) was used to calibrate the oxygen analyser and pure stock nitrogen was used for zero calibration. We recorded O_2_ concentration and temperature within the chamber at 1-s intervals. Oxygen consumption was calculated according to Hill [52] on the basis of the lowest 10-min average of O_2_ consumption. To make measurements comparable with the above-cited studies, we used a respiratory quotient of 0.70 and an energy equivalent of 20 kJ l^−1^ O_2_. Likewise, reported body masses correspond to those measured just before the beginning of the metabolic measurements.

### Metabolic and Climatic Data

BMR (W) and body mass (*m*_b_; g) data of 39 species of non-breeding shorebirds were measured (see above) or compiled from the literature (Appendix S1). All birds belonged to migratory species that breed in the Northern Hemisphere. BMR data were included only if birds were measured under standardized conditions (i.e. within the thermoneutral zone under postabsorptive digestive conditions during the resting phase of the daily cycle on resting, non-growing, non-reproductive birds; [4]). Moreover, all BMRs represent wild-caught birds, as there is evidence that BMR in birds raised in captivity differs from their wild-caught counterparts [53]. Given these criteria, several BMR data for the following four species were not included in the analyses: Eurasian woodcock *Scolopax rusticola* [51], American woodcock *Scolopax minor* [51], little stint *Calidris alba* [54], and stone curlew *Burhinus oedicnemus* [42]. We included BMR values for four outdoor-captive species of shorebirds that had been captured at the Dutch Wadden Sea (red knot *Calidris canutus*, Eurasian oystercatcher *Haemotopus ostralegus*, grey plover *Pluvialis squatarola*, and turnstone *Arenaria interpres*) because they were housed under natural temperature and photoperiodic regimes and their BMR did not differ substantially from other individuals captured at similar latitudes [27]. We opted to include data irrespective of sample size (including data that represented only one individual), since this approach substantially increased the “full dataset” (see below) and did not change any of the conclusions (results not shown, but analyses can be made based on the data in the Appendix S1). For several species, metabolic data were available from more than one study or geographic area, and hence different climatic conditions. In this case, we represented them with multiple points (e.g. [55]). Study locations, dates, and the corresponding latitude-longitude coordinates were obtained from the original articles and maps. Birds were captured at geographical locations ranging from 33°S to 76°N. This allowed us to examine the relationship between BMR and various climatic variables that are known to be major contributors to BMR variation in (shore)birds: latitude (°N or °S), ambient temperature [mean, minimum, and maximum temperature (°C)], solar radiation (W m^−2^), and windspeed (m s^−1^) (e.g. [31], [39], [56]). These variables, except latitude, are monthly averaged meteorological data from 10 to 22 years (1983−2005) obtained from the Surface Meteorology and Solar Energy (SSE) project (http://eosweb.larc.nasa.gov/sse/) [57]. Because temperatures, latitude and solar radiation were highly correlated (Pearson correlation matrix: all absolute values of *r* ≥0.67, *P*<0.01), we reduced the six climatic variables using principal component analysis, so that they would not display multicolinearity. Components with eigenvalues >1 were retained and their scores were used in subsequent analysis (e.g. [58]).

Each shorebird species was classified as ‘coastal’ or ‘inland’ according to their main non-breeding habitat occupancy [49], [50]. Because changes in body composition during migration can substantially affect shorebirds’ BMR (e.g. [59], [60]), we further classified individuals as migrating (individuals captured and measured during pre-migratory and migration periods) or wintering (individuals captured and measured in their wintering grounds). Therefore, we conducted analyses using all individuals (“full dataset”) or a subset including wintering individuals only (“wintering dataset”).

### Interspecific Analyses

A critical consideration in comparative analyses is the need to control for non-independence in the data due to phylogeny [61]–[63]. However, the use of phylogenetically-controlled methods has been questioned when applied to the study of metabolic traits such as BMR (e.g. [9]). We therefore performed both ordinary (i.e. non-phylogenetic) least squares (OLS) and phylogenetic generalized least squares (PGLS) approaches.

As a first approximation to assess the potential effect of habitat occupancy, we used a general linear model (GLM, no phylogenetic control) with the categorical variables ‘habitat occupancy’ (coastal *vs* inland) and ‘migratory status’ (migrating *vs* wintering, in the full dataset only) as fixed factors and the continuous variables ‘body mass’ and ‘climatic component’ (CC1; see *Results*). For these conventional analyses, we used STATISTICA7.0 (StatSoft Inc., Tulsa, OK, USA).

**Table 1:** Scores of a principal component analysis on climatic variables for the “full” and “wintering” datasets (see *Materials and methods* for details).

Climatic variable	Component 1 (full dataset)	Component 1 (wintering dataset)
Latitude	0.88	0.95
Mean temperature	−0.99	−0.98
Minimum temperature	−0.97	−0.98
Maximum temperature	−0.96	−0.98
Solar radiation	−0.92	−0.97
Windspeed	0.33	0.19
Eigenvalue (% variation explained)	4.62 (76.95%)	4.78 (79.72%)

To account for the possible effects of phylogenetic inertia –the tendency of closely related species to resemble each other– we assessed the strength of the phylogenetic signal in BMR, *m*_b_, mass-adjusted BMR, and CC1 using the randomization test for the mean-squared error as described in Blomberg et al. ([64]; Matlab program PHYSIG_LL.m). We also calculated the *K* statistics as a measure of the amount of signal [64]. To construct our phylogeny, we used the Thomas et al.’s supertree of shorebirds [65], which was trimmed to include only the 39 species for which BMR and *m*_b_ data were available. Those species measured in multiple locations were included by adding them as polytomies to the appropriate species tip in the trimmed tree (full dataset phylogeny; Appendix S2). Additionally, this phylogenetic tree was re-trimmed to include a restricted sample of 25 species of wintering shorebirds (wintering dataset phylogeny; Appendix S2). Branch lengths were specified by Pagel’s [66] arbitrary method. Both phylogenetic trees were edited and then saved as phylogenetic variance-covariance matrices using Mesquite [67]. Data for BMR, *m*_b_ and mass-adjusted BMR were log_10_-transformed prior to all analyses.

**Table 2:** Statistics for randomization tests for significance of phylogenetic signal for log *m*_b_, log BMR, log mass-adjusted BMR, and CC1 for either the **A** 39 species used in the “full dataset” or the **B** 25 species used in the “wintering dataset”.

Trait	Expected MSE_0_/MSE	Observed MSE_0_/MSE	*K*	MSE_candidate_	MSE_star_	*P*	ln ML_candidate_	ln ML_star_
**A** Full dataset								
log *m*_b_	4.696	8.793	1.873	0.027	0.154	<0.001	36.452	−44.106
log BMR	4.696	4.640	0.988	0.020	0.065	<0.001	49.320	−4.433
log BMR/*m*_b_^0.624^	4.696	0.501	0.107	0.010	0.005	0.789	80.948	113.367
CC1	4.696	0.594	0.126	1.725	1.000	0.200	−155.083	−130.04
**B** Wintering dataset								
log *m*_b_	2.534	3.304	1.309	0.051	0.133	<0.001	3.272	−15.478
log BMR	2.524	2.332	0.924	0.035	0.068	<0.001	10.557	−2.273
log BMR/*m*_b_^0.685^	2.362	0.489	0.197	0.011	0.005	0.855	33.744	47.693
CC1	2.682	0.538	0.213	1.880	1.000	0.680	−67.144	−54.8321

The tip data and phylogenetic trees are shown in Appendices S1 and S2, respectively. Significant results for the randomization test of the mean squared error (MSE; lower values indicate better fit of tree to data) on the phylogenetic tree indicate the presence of phylogenetic signal for all traits. *K* statistics indicate the amount of phylogenetic signal relative to a Brownian motion expectation [64].

Abbreviations: *m*_b_, body mass; BMR, basal metabolic rate; CC1, climatic component (see *Materials and methods*); MSE, mean squared error; ML, maximum likelihood.

The “candidate” is the observed tree topology, whereas the “star” is a tree with all branch lengths set to one.

Mass-adjusted BMR was calculated as according to the equation: mass-adjusted BMR  =  BMR/*m*_b_ *^b^*, where *b* represents the slope of the OLS regression of log BMR on log *m*_b_ for all species combined in each dataset (see Table 3).

Next, we performed multiple regressions using the Matlab REGRESSIONv2.m program [68]. We used three of the models available in REGRESSIONv2.m, including conventional OLS regressions (assumes a star phylogeny), phylogenetic generalized least squares (PGLS; assumes given branch lengths) and an Ornstein-Uhlenbeck transformation (Reg_OU_). First, we used the full dataset to identify significant correlates of BMR. These models included a simple linear regression of log_10_ BMR on log_10_ *m*_b_ and various multiple regressions with independent variables of log *m*_b_, EC1, migratory status (0 =  migrating, 1 =  wintering), and habitat occupancy (0 =  coastal, 1 =  inland). Next, we performed the same type of analyses for the wintering dataset, which excluded data from individuals measured outside the winter period and thus contained no variable on migratory status. To determine the best fit model for multiple-regression data, we used log-likelihood ratio tests (LRTs) and the Akaike Information Criterion in both its original (AIC) and corrected (AIC_c_) forms [69], [70]. We considered the best-fit model as that model with the lowest AIC score and the highest log maximum likelihood [68], [71]. As a rule of thumb, models whose AIC is ≤2 units larger can also be said to have substantial support [69], [70]. Moreover, we used partial *F*-tests to determine which independent variables significantly influenced log BMR. Statistical significance was accepted at *P*<0.05 for all tests.

**Figure 1:**
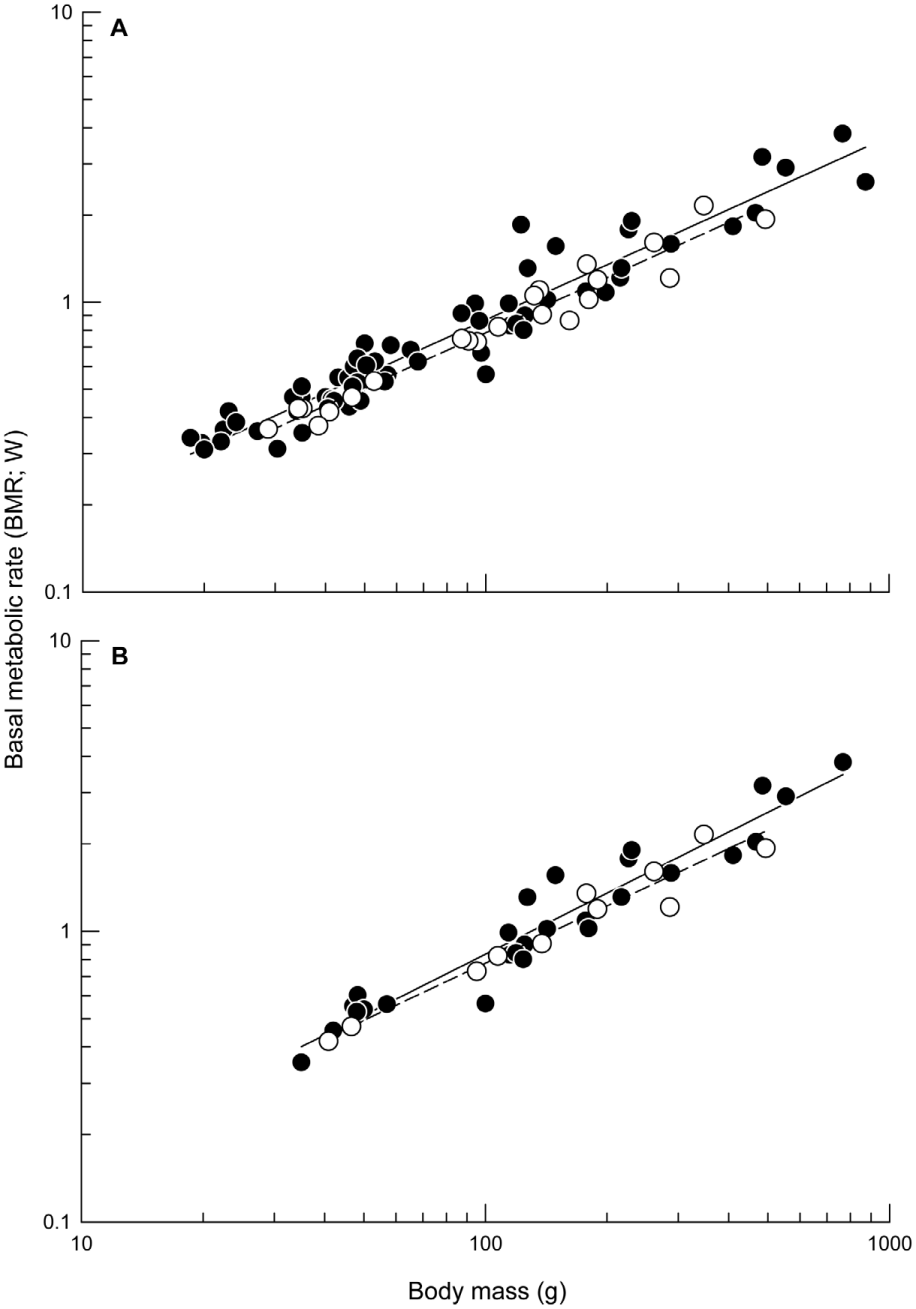
Shorebirds’ basal metabolic rate (BMR) increased consistently with body mass. Relationship between BMR and body mass for coastal (black circles, solid regression line) and inland shorebirds (open circles, dashed regression line) in the **A** “full dataset” and the **B** “wintering dataset”. Regression lines were obtained with conventional (i.e. non-phylogenetically independent) regressions. In **A,** regression equations were: log BMR  = −1.325+0.632 log *m*_b_ and log BMR  = −1.368+0.632 log *m*_b_ for coastal (N = 70) and inland shorebirds (N = 22), respectively. In **B,** regression equations were: log BMR  = −1.481+0.702 log *m*_b_ and log BMR  = −1.419+0.655 log *m*_b_ for coastal (N = 28) and inland shorebirds (N = 11), respectively.

**Figure 2:**
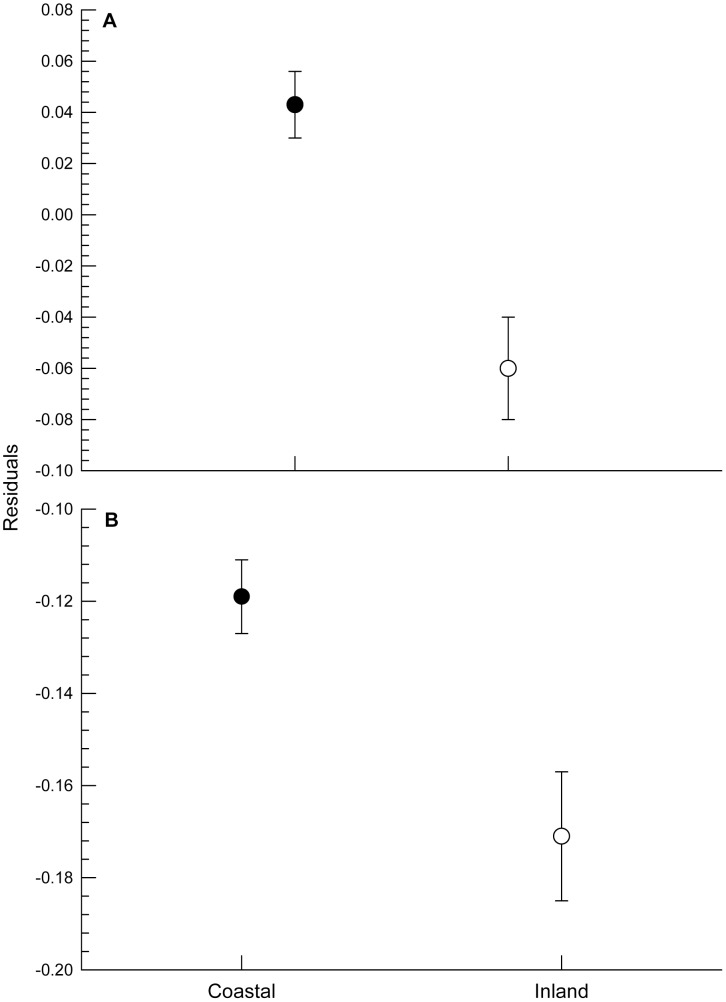
Basal metabolic rate (BMR) was higher in coastal than inland shorebirds. Average residuals (±SE) from the GLM analyses for coastal (black circles) and inland (open circles) shorebirds in the **A** “full dataset” and the **B** “wintering dataset” after controlling for body mass, migratory status and climatic conditions. Note that scale in **A** is different from that in **B.**

### Intraspecific Analyses

Comparisons of closely related taxa (such as populations, subspecies, or species) from different environments provide the opportunity for more detailed examination of physiological adjustments to environmental conditions without the potentially confounding effects of phylogeny. We compared the observed BMR values for each of the 12 species wintering in temperate inland wetlands to those predicted from the allometric equations for shorebirds wintering in temperate [27] and tropical coastal areas [29]. Observed and predicted BMRs were compared using paired *t*-tests. BMRs for three species we captured in temperate inland habitats (the dunlin, the Eurasian curlew, and the common ringed plover *Charadrius hiaticula*) had been previously measured in the coast of Guinea-Bissau (West Africa; [29]), so their mass-specific BMRs were compared.

**Table 3:** Partial regression coefficients and *P* values from phylogenetically informed and conventional ordinary least squares (OLS) multiple regressions.

Model	Log *m*_b_ a (SE)	CC1 (SE)	CC1 *P*	Migratory status *P*	Habitat *P*	Ln ML^b^	Transform parameter	*R* ^2^	SEE	*P* for LTR_S_ *vs* OLS full model^c^	AIC	AIC_C_
Full dataset												
OLS	0.624 (0.019)	–	–	–	–	113.442	–	0.923	0.071	0.003	−220.844	−220.571
OLS	0.639 (0.020)	0.017 (0.008)	0.035	–	–	115.739	–	0.927	0.070	0.009	−223.477	−223.017
OLS	0.650 (0.021)	0.013 (0.009)	0.136	0.343	–	116.323	–	0.928	0.070	0.004	−222.647	−221.949
**OLS** d	**0.651 (0.019)**	**0.021 (0.008)**	**0.007**	–	**0.004**	**120.017**	–	**0.933**	**0.067**	**0.345**	−**230.035**	−**229.337**
OLS full model	0.659 (0.022)	0.018 (0.008)	0.038	0.360	0.005	120.463	–	0.934	0.067	–	−228.925	−227.937
PGLS	0.614 (0.065)	–	–	–	–	81.012	–	0.498	0.101	–	−156.024	−155.751
PGLS	0.586 (0.063)	0.028 (0.008)	0.005	–	–	85.170	–	0.541	0.097	–	−162.339	−161.879
PGLS	0.589 (0.069)	0.024 (0.009)	0.014	0.916	–	85.175	–	0.541	0.098	–	−160.351	−159.653
PGLS	0.597 (0.063)	0.024 (0.008)	0.003	–	0.182	85.105	–	0.551	0.097	–	−162.211	−161.513
PGLS	0.600 (0.069)	0.023 (0.009)	0.010	0.916	0.185	86.111	–	0.551	0.098	–	−160.222	−159.234
Reg_OU_	0.624 (0.019)	–	–	–	–	113.422	1.301e-17	0.923	0.071	–	−218.844	−218.384
Reg_OU_	0.639 (0.020)	0.017 (0.008)	0.035	–	–	115.739	1.301e-17	0.927	0.070	–	−221.477	−220.780
Reg_OU_	0.649 (0.019)	0.013 (0.009)	0.136	0.292	–	116.323	1.301e-17	0.928	0.070	–	−220.647	−219.659
Reg_OU_	0.651 (0.019)	0.021 (0.008)	0.007	–	0.004	120.017	1.301e-17	0.933	0.067	–	−228.035	−227.047
Reg_OU_	0.659 (0.021)	0.018 (0.008)	0.038	0.360	0.005	120.463	1.301e-17	0.934	0.067	–	−226.925	−225.592
Wintering dataset												
OLS	0.685 (0.033)	–	–	–	–	47.725	–	0.923	0.073	0.004	−89.450	−88.764
OLS	0.681 (0.033)	0.011 (0.012)	0.354	–	–	48.197	–	0.925	0.073	0.002	−88.393	−87.217
**OLS** d **full model**	**0.679 (0.029)**	**0.042 (0.014)**	**0.006**	–	**0.003**	**53.183**	–	**0.942**	**0.068**	–	−**96.366**	−**94.548**
PGLS	0.692 (0.075)	–	–	–	–	33.777	–	0.696	0.104	–	−61.554	−60.868
PGLS	0.647 (0.071)	0.032 (0.012)	0.009	–	–	37.519	–	0.749	0.096	–	−67.039	−65.862
PGLS	0.629 (0.058)	0.058 (0.013)	<0.001	–	<0.001	42.965	–	0.837	0.079	–	−81.929	−80.111
Reg_OU_	0.684 (0.033)	–	–	–	–	47.725	1.301e-17	0.923	0.073	–	−87.450	−86.274
Reg_OU_	0.681 (0.033)	0.012 (0.012)	0.354	–	–	48.197	1.301e-17	0.925	0.073	–	−86.393	−84.575
Reg_OU_	0.674 (0.032)	0.047 (0.013)	0.001	–	<0.001	53.361	1.301e-17	0.931	0.065	–	−94.723	−92.098

Log basal metabolic rate (BMR) was the dependent variable, and various combinations of log body mass (*m*_b_), climatic component (CC1; see *Materials and methods*), migratory status (0 =  migration period, 1 =  winter period; note that this category was not included in the “wintering dataset” models), and habitat (0 =  coastal, 1 =  inland), were independent variables. Phylogenetic multiple regressions included three of the models available in the Matlab REGRESSIOMv2.m program, including OLS regressions, phylogenetic generalized least squares (PGLS) and an Ornstein-Uhlenbeck transformation (Reg_OU_). *R* ^2^ values are not comparable between OLS and phylogenetic multiple regressions [68].

a Significant at *P*<0.0001 for all models, so *P* values are not included in the table.

b ML, maximum likelihood.

c Ln likelihood-ratio tests (LRT) compare fit of the full OLS model including all candidate independent variables with the fit of reduced models. Twice the difference in the log likelihoods is asymptotically distributed as a χ^2^ with degrees of freedom equal to the difference in the number of parameters in the two models. A *P* value <0.05 indicates that the reduced model has a significantly worse fit to the data than the full model.

d Best models based on the lowest Akaike’s information criterion (AIC) and lowest standard error of the estimate (SEE).

Based on the data for dunlins captured at Cádiz Bay Natural Park (N = 10; coastal habitat) and those captured in the Extremadura rice fields (N = 10; inland habitat) during the same period of the year (February-March 2010, 25 days between captures), it was possible to investigate whether BMR differed between individuals wintering in an inland freshwater habitat and individuals wintering in a coastal saline habitat at similar latitudes (<3° between capture sites, ∼300 km). We assumed that individuals from both inland and coastal localities belonged to the subspecies *C. alpina alpina* [43], [50]. Because we used monthly averaged values in order to estimate the main climatic features of each location (thus climate variables showed no variance), we used GLM analysis with *m*_b_ as the covariate and habitat occupancy (coastal *vs* inland) as the categorical factor.

**Table 4:** Body mass (g) and basal metabolic rate (BMR; W) for 12 species of shorebird wintering in temperate inland habitats (Spain; this study), and BMR predicted by allometric equations for shorebirds wintering in temperate (the Netherlands; Kersten & Piersma 1987) and tropical coasts (West Africa; Kersten et al. 1998). BMR (in brackets) expressed as a percentage of predicted values.

Species		N	Body mass (SE)	Observed BMR (SE)	Predicted BMR Kersten & Piersma (1987) (% of predicted values)	Predicted BMR Kersten *et al.* (1998) (% of predicted values)
stone curlew	*Burhinus oedicnemus*	8	493.73 (14.01)	1.93 (0.17)	3.02 (62.15)	2.41 (77.96)
Eurasian golden plover	*Pluvialis apricaria*	6	189.23 (4.90)	1.19 (0.10)	1.50 (79.15)	1.20 (98.81)
common ringed plover	*Charadrius hiaticula*	3	56.10 (3.75)	0.53 (0.02)	0.62 (85.53)	0.50 (106.12)
little ringed plover	*Charadrius dubius*	15	40.86 (0.96)	0.42 (0.02)	0.49 (85.40)	0.40 (105.79)
common snipe	*Gallinago gallinago*	4	95.15 (3.12)	0.72 (0.08)	0.91 (79.05)	0.73 (98.34)
black-tailed godwit (m)	*Limosa limosa*	5	261.26 (8.74)	1.60 (0.04)	1.90 (84.12)	1.52 (105.18)
black-tailed godwit (f)	*Limosa limosa*	4	347.15 (10.63)	2.15 (0.32)	2.34 (91.89)	1.87 (115.05)
Eurasian curlew	*Numenius arquata*	1	875	2.60	4.59 (56.64)	3.65 (71.24)
spotted redshank	*Tringa erythropus*	1	137.90	0.91	1.19 (76.23)	0.96 (95.01)
common sandpiper	*Actitis hypoleucos*	3	46.55 (3.25)	0.47 (0.11)	0.54 (86.90)	0.44 (107.72)
little stint	*Calidris minuta*	5	30.36 (1.78)	0.31 (0.03)	0.40 (78.27)	0.32 (96.82)
dunlin	*Calidris alpina*	41	45.79 (0.81)	0.44 (0.01)	0.53 (82.34)	0.43 (102.05)
ruff (m)	*Philomachus pugnax*	10	177.89 (4.43)	1.35 (0.05)	1.44 (93.93)	1.15 (117.22)
ruff (f)	*Philomachus pugnax*	12	107.30 (6.27)	0.82 (0.05)	0.99 (82.48)	0.80 (102.67)

Note: m, males; f, female.

The predicted BMR was calculated as the average of individual values for each species.

## Results

### Climatic Variables

Data on latitude, temperature, solar radiation, and windspeed patterns were simplified using PCA analysis, either for the full dataset or the wintering dataset. In both cases, PCA produced one main component (CC1) with eigenvalues of 4.62 and 4.78, respectively. Both components were mainly related with temperature, and accounted for 76.96% (full dataset) and 79.72% (wintering dataset) of the total variance (Table 1).

**Figure 3:**
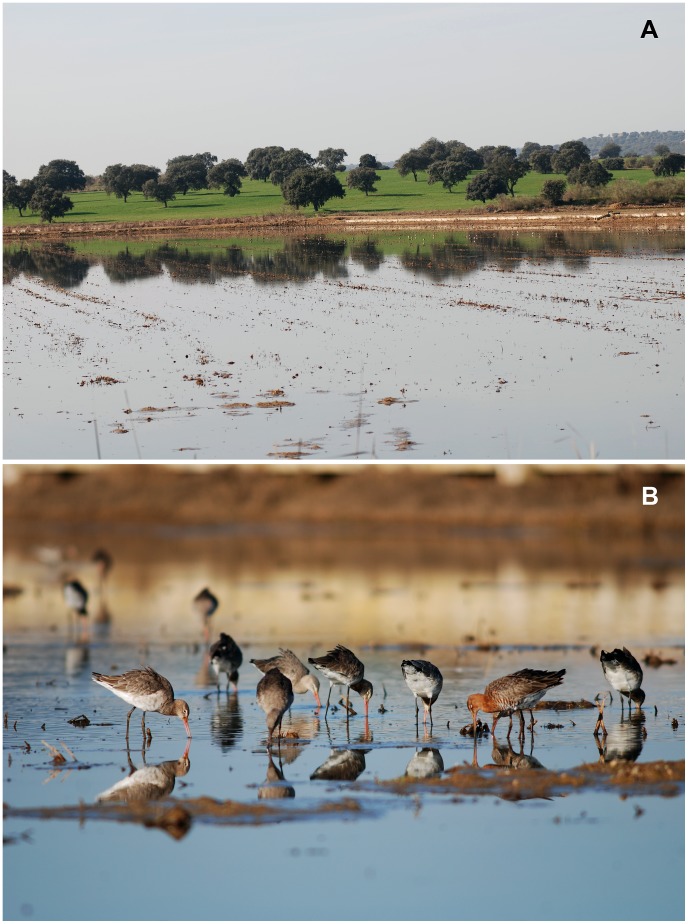
Large numbers of shorebirds consistently use temperate inland freshwater habitats during winter and migration. **A** Shorebirds in inland freshwater wetlands, such as rice fields in Extremadura, often experience favourable feeding conditions (absence of tidally-induced food restrictions, low salinity of prey and drinking water, and less windy microclimates) which could contribute to reduce the levels of energy expenditure, and hence BMR. **B** Black-tailed godwits feeding on spilled rice seeds in Extremadura’s rice fields.

### Phylogenetic Signal

When tested individually, BMR and *m*_b_ had a strong and significant phylogenetic signal (Table 2). In contrast, mass-adjusted BMR did not retain a significant phylogenetic pattern (Table 2). This suggests that the strong phylogenetic history in *m*_b_ (*K* values higher than 1) accounted for the signal in BMR. Nor did we find any significant phylogenetic signal in CC1, irrespective of the dataset used (*P*>0.05 in both cases; Table 2).

### Interspecific Comparisons

BMR increased consistently and strongly with *m*_b_ regardless of the method and the dataset used (*P*<0.0001; Figure 1). The GLM analyses indicated the BMR of inland shorebirds was lower than that of coastal shorebirds when considering all data tips (*F*_1, 89_ = 6.47, *P* = 0.01; Figure 1A), but not when considering the data tips for wintering shorebirds only (*F*_1, 36_ = 2.27, *P* = 0.18; Figure 1B). However, the presence or absence of significant differences between coastal and inland shorebirds’ BMR could be due to various biases, such as the migratory status (in the case of the full dataset) or composition of samples from different sites. When controlling for the climatic conditions by adding the climatic component (CC1) as a predictive variable, inland shorebirds’ BMR was lower than that of coastal shorebirds either in the full dataset (*F*_1, 88_ = 9.34, *P* = 0.003; Figure 2A) or the wintering dataset (*F*_1, 35_ = 10.14, *P* = 0.003; Figure 2B).

Multiple conventional and phylogenetic regression models confirmed these results, showing that *m*_b_, CC1 and non-breeding habitat occupancy were significant predictors of a shorebird’s BMR (Table 3). The low (close to zero) transform parameters indicate that a star phylogeny (i.e. the OLS model) best fit the data (Table 3).

When considering all the data tips (i.e. the full dataset), the best fit was provided by an OLS model that included log *m*_b_, CC1, and habitat occupancy (coded as one dummy variable) as independent variables, and all of these contributed significantly to explain the variability in BMR (Table 3). Based on LRTs and AIC, the models that included migratory status performed significantly worse than those that did not (Table 3). Again, when considering wintering individuals only (i.e. the wintering dataset), the full model (OLS), which included log *m*_b_, CC1, and habitat occupancy produced the best fit (Table 3).

### Intraspecific Comparisons

All observed BMR values in inland shorebirds were below those predicted from the allometric equation for shorebirds wintering in temperate coasts (Table 4; *t*_13_ = −2.50; *P* = 0.03), but did not differ significantly from allometrically-predicted values for tropical winterers (Table 4; *t*_13_ = 0.79; *P* = 0.44). When comparing the mass-specific BMR observed in three ringed plovers and a single Eurasian curlew, we found that they were 16% and 40% lower, respectively, than that observed in individuals wintering in Guinea-Bissau [29].

At a similar latitude, BMR of inland dunlins was on average 15% lower than in coastal dunlins (GLM: *F*_1, 17_ = 4.56, *P* = 0.047), despite the warmer climate at the coastal site (mean temperature  = 9.6°C and 16.2°C, respectively; Appendix S1). Compared to a single dunlin wintering along the tropical coast of Guinea-Bissau [29], temperate inland dunlins still had a lower mass-adjusted BMR (10.1 and 9.5 W kg^−1^, respectively).

## Discussion

In this study, we tested the metabolic dichotomy between marine and non-marine habitats using migratory shorebirds as a model. We hypothesized that shorebirds using inland freshwater habitats during the non-breeding season would have a reduced BMR compared with those shorebirds occupying coastal saline habitats during this season. Based on ordinary and phylogenetic least squares models (OLS and PGLS, respectively), we present empirical evidence supporting the notion that inland shorebirds have a lower BMR than coastal shorebirds. This pattern is consistent with previous observations that inland shorebirds species have lower BMR than expected on the basis of body mass [40]–[42]. Conventional OLS models consistently provided the best fit for the data in this study, indicating no phylogenetic signal (see *Results*). These models indicated that migratory shorebirds’ BMR has a relatively low phylogenetic inertia (i.e. a lack of phylogenetic signal for mass-adjusted values), which is in agreement with the observation that BMR in migrants shows a lower phylogenetic signal than in non-migrants [12]. Several types of “errors” are known to obscure the phylogenetic signals, including phenotypic plasticity and measurement of error of various types [64]. Errors in branch lengths definitely exist in our study given that we used arbitrary values. Nevertheless, OLS and PGLS models produced essentially the same results.

What proximate mechanisms might underlie a reduced BMR in inland shorebirds? Vander Haegen [40] argued that one possibility is that inland shorebirds migrate considerably shorter distances than coastal shorebirds, which probably do not incur as high energetic demands during the pre-migratory [28], [39] and migration periods [30]. However, coastal shorebirds typically show low BMR levels while wintering in the tropics [29], suggesting that distance travelled between breeding and non-breeding grounds is not the primary cause for low BMR in inland shorebirds. This suggests that climate and/or habitat type, and not migration distance, may be a primary cause for low BMR in inland shorebirds [42]. Inland shorebird species live in non-tidal habitats more protected from wind than coastal species (see Figure 3), and may subsequently reduce heat loss by convection during periods of low environmental temperatures [41], [42], [56]. Duriez et al. [41], for example, showed that the energetic requirements of Eurasian woodcocks *Scolopax rusticola* (a forest-dwelling shorebird) were lower than those of coastal shorebirds living in windy unsheltered habitats, which may lead to their low BMR (40% lower than predicted from the allometric equation for shorebirds wintering in temperate coasts; [27]). A lower-than-expected BMR was also found in another inland shorebird, the Eurasian curlew [42], which suggests that this could be an adaptation to sheltered terrestrial habitats. On the other hand, Wiersma & Piersma [56] showed that red knots occurring in open coastal habitats experience much greater heat losses than expected on the simple basis of air temperature alone. This highlights the importance of simultaneously considering both weather conditions and habitat features in inter- and intraspecific comparisons of avian energetics. Our analyses indicate that inland species still have lower BMR than coastal ones after correcting for potentially explanatory factors such as climate (including windspeed, air temperature and solar radiation) and migratory status.

Inland shorebirds whose foraging patterns are not restricted by the tides may afford to eat many small meals throughout day, which should spread the so-called ‘heat increment of feeding’ –the increase in resting metabolic rate observed after ingestion of a meal, associated with heat production during the processes of digestion, assimilation and nutrient interconversion [72]– over longer periods and thus contribute to offset thermoregulatory demands while under sub-thermoneutral conditions [72], [73]. Instead, enforced rest at high tide might reduce opportunities for coastal shorebirds to use the heat increment of feeding, especially in small-bodied species that completely empty themselves out before a new high-falling tide returns. This could imply that the heat increment of feeding (and associated locomotion/foraging activity) will have a greater compensatory effect on the metabolic rate during cold exposure in inland shorebirds than in coastal ones, thus allowing lower metabolic costs.

Low BMR might also be associated with scarcity and unpredictability of food [14], [74]. There is no doubt that coastal marine habitats are more predictable and productive than inland freshwater habitats [75]. Thus, inland species (or populations) that have evolved in less productive environments may have a slower pace of life compared with coastal representatives in the presence of abundant food. In addition, as outlined above, many shorebirds foraging on intertidal areas are regularly forced to rest at high tide, so they have only a limited time to find their food and meet their high energy requirements (especially during cold-temperate winters or migratory periods). To cope with this time constraint, they probably increase their rate of food intake while simultaneously increasing the mass of their digestive organs [38], which may increase BMR as these organs are relatively costly to maintain [76], [77]. This potentially enables migrating coastal shorebirds to metabolize energy at rates of up to ten times BMR [78]. Higher BMR may then improve the efficiency of food digestion, which would be advantageous for coastal shorebirds under situations of intermittent (but predictable) feeding routines.

Another possible non-mutually exclusive factor contributing to the lower BMR of inland shorebirds species compared to coastal ones concerns the ionic composition of their diets. From an osmoregulatory standpoint, inland shorebirds that consume low-salt content diets will not need to invest so much in osmoregulation as compared with coastal shorebirds, which generally feed on marine invertebrates that are isosmotic (and isotonic) with seawater [43], [79], [80]. Gutiérrez et al. [43] recently showed that the BMR of dunlins increased by 17% during seawater acclimation. This is in agreement with the present observation that coastal dunlins have a mass-specific BMR 15% higher than their coastal congeners. Low salinity levels in inland waters may thus partly explain the low BMR of inland shorebirds.

Differences in BMR between coastal and inland shorebirds may reflect phenotypic plasticity, genotypic adaptation, or to some interaction between these mechanisms. Phenotypic plasticity seems to be important in shorebirds facing energy constraints, as suggested by differences in BMR between coastal and inland dunlins. Nevertheless, our interspecific analyses accounted for the influence of climatic and organismal factors (phenotypic variation), suggesting that part of the variation in basal metabolism between both groups might be attributed to genetic (evolutionary) change [3], [81]. Evidence for genotypic divergence in metabolic traits, however, could only be demonstrated by conducting common-garden experiments that eliminate phenotypic plasticity as a source of phenotypic variation (e.g. [18], [82], [83]). At this point, we suspect that natural selection has influenced the basal metabolism among inland shorebirds. Further research, including BMR measurements from a wider variety of inland shorebirds, is needed to determine if these general patterns are robust.

The existence of a metabolic dichotomy could have important implications for comparative studies, since comparisons of the energetic traits of marine/coastal birds with those of non-marine birds may lead to misleading conclusions regarding physiological adaptation.

## Supporting Information

Appendix S1 Data on body mass (Mass; g), basal metabolic rate (BMR; W), habitat (0 =  coastal, 1 =  inland), migratory status (Status; 0 =  migratory period, 1 =  wintering period), latitude (N-S), longitude (E-W), radiation (W m^−2^), mean temperature (*T*_mean_; °C), minimum temperature (*T*_min_; °C), maximum temperature (*T*_max_; °C), and windspeed (Wind; ms^−1^) for shorebirds species in this study. Asterisks indicate data included in the “wintering dataset”.
(RTF)

Appendix S2 Phylogeny for the **A** 39 species (92 tips) of shorebirds included in the “full dataset” and for the **B** 25 species (39 tips) included in the “wintering dataset”. Both trees were derived from the shorebird supertree developed by Thomas et al. [1]. Branch lengths specified by Pagel’s [2] arbitrary method.
(RTF)
