# Supplementary material for: Avian BMR in Marine and Non-Marine Habitats: A Test Using Shorebirds
Source: PLoS One. 2012 Jul 31;7(7):e42206. doi: 10.1371/journal.pone.0042206 (PMC3409136; doi:10.1371/journal.pone.0042206)
Supplement: Appendix S1 — Data on body mass (Mass; g), basal metabolic rate (BMR; W), habitat (0 = coastal, 1 = inland), migratory status (Status; 0 = migratory period, 1 = wintering period), latitude (N-S), longitude (E-W), radiation (W m−2), mean temperature (T mean; °C), minimum temperature (T min; °C), maximum temperature (T max; °C), and windspeed (Wind; ms−1) for shorebirds species in this study. Asterisks indicate data included in the “wintering dataset”. (RTF) [file pone.0042206.s001.rtf]

Appendix S1. Data on body mass (Mass; g), basal metabolic rate (BMR; W), habitat (0 = coastal, 1 = inland), migratory status (Status; 0 = migratory period, 1 = wintering period), latitude (N-S), longitude (E-W), radiation (W m-2), mean temperature (Tmean; ºC), minimum temperature (Tmin; ºC), maximum temperature (Tmax; ºC), and wind speed (Wind; ms-1) for shorebirds species in this study. Asterisks indicate data included in the “wintering dataset”.
Species	N	 Mass	BMR	Habitat	Status	Latitude 	Longitude 	Radiation	Tavg 	Tmin	Tmax	Wind	Source	
Haemotopus ostralegus*	1	410.00	1.83	0	1	11º40'N	15º45'W	265.44	31.07	23.37	41.03	3.52	[1]	
Haemotopus ostralegus*	4	554.00	2.91	0	1	53º11'N	6º36'E	125.01	15.00	5.00	10.00	2.00	[2]	
Burhinus oedicnemus*	8	493.73	1.93	1	1	39º01'N	5º58'W	96.26	8.27	4.92	13.50	3.81	[3]	
Pluvialis apricaria*	6	189.23	1.19	1	1	39º01'N	5º58'W	132.09	9.60	5.33	16.10	3.99	[3]	
Pluvialis apricaria	1	161.00	0.86	0	0	56º12'N	16º24'E	200.85	17.80	15.30	20.60	3.95	[4]	
Pluvialis fulva*	12	127.00	1.31	0	1	21º24N	157º58W	246.69	23.90	23.90	23.90	6.41	[5]	
Pluvialis squatarola*	2	177.00	1.09	0	1	11º40'N	15º45'W	265.44	31.07	23.37	41.03	3.52	[1]	
Pluvialis squatarola*	3	226.00	1.78	0	1	53º11'N	6º36'E	125.01	15.00	5.00	10.00	2.00	[2]	
Pluvialis squatarola*	4	230.00	1.91	0	1	33º05S	18º02E	243.35	22.80	16.37	33.00	4.88	[1]	
Charadrius hiaticula*	2	48.00	0.53	0	1	11º40'N	15º45'W	265.44	31.07	23.37	41.03	3.52	[1]	
Charadrius hiaticula	3	56.10	0.53	1	1	39º01'N	5º58'W	96.26	8.27	4.92	13.50	3.81	[3]	
Charadrius hiaticula	6	47.00	0.51	0	0	56º12'N	16º24'E	200.85	17.80	15.30	20.60	3.95	[4]	
Charadrius dubius*	15	40.87	0.42	1	1	39º01'N	5º58'W	96.26	8.27	4.92	13.50	3.81	[3]	
Charadrius dubius	1	29.00	0.37	0	0	56º12'N	16º24'E	200.85	17.80	15.30	20.60	3.95	[4]	
Charadrius alexandrinus*	2	42.00	0.46	0	1	11º40'N	15º45'W	265.44	31.07	23.37	41.03	3.52	[1]	
Scolopax rusticola*	28	286.00	1.21	1	1	46º09'N	0º24'W	58.75	8.68	5.03	13.30	8.81	[6]	
Gallinago gallinago*	4	95.15	0.73	1	1	39º01'N	5º58'W	132.09	9.60	5.33	16.10	3.99	[3]	
Gallinago gallinago	6	91.00	0.73	0	0	56º12'N	16º24'E	200.85	17.80	15.30	20.60	3.95	[4]	
Limosa limosa (f)*	4	347.15	2.15	1	1	39º01'N	5º58'W	132.09	9.60	5.33	16.10	3.99	[3]	
Limosa limosa (m)*	5	261.26	1.60	1	1	39º01'N	5º58'W	132.09	9.60	5.33	16.10	3.99	[3]	
Limosa lapponica	1	215.00	1.21	0	0	56º12'N	16º24'E	200.85	17.80	15.30	20.60	3.95	[4]	
Limosa lapponica (f)*	6	288.00	1.59	0	1	11º40'N	15º45'W	265.44	31.07	23.37	41.03	3.52	[1]	
Limosa lapponica (m)*	3	217.00	1.31	0	1	11º40'N	15º45'W	265.44	31.07	23.37	41.03	3.52	[1]	
Numenius phaeopus*	4	467.00	2.03	0	1	11º40'N	15º45'W	265.44	31.07	23.37	41.03	3.52	[1]	
Numenius phaeopus*	2	485.00	3.17	0	1	33º05S	18º02E	243.35	22.80	16.37	33.00	4.88	[1]	
Numenius arquata*	1	767.00	3.82	0	1	11º40'N	15º45'W	265.44	31.07	23.37	41.03	3.52	[1]	
Numenius arquata	1	875.00	2.60	1	1	39º01'N	5º58'W	132.09	9.60	5.33	16.10	3.99	[3]	
Tringa erythropus*	1	137.90	0.91	1	1	39º01'N	5º58'W	132.09	9.60	5.33	16.10	3.99	[3]	
Tringa totanus*	3	124.00	0.80	0	1	11º40'N	15º45'W	265.44	31.07	23.37	41.03	3.52	[1]	
Tringa totanus	5	96.00	0.86	0	0	56º12'N	16º24'E	200.85	17.80	15.30	20.60	3.95	[4]	
Tringa totanus*	6	149.00	1.56	0	1	56º00'N	2º35'E	48.75	1.83	-0.96	5.01	6.38	[7]	
Tringa nebularia*	2	180.00	1.02	0	1	11º40'N	15º45'W	265.44	31.07	23.37	41.03	3.52	[1]	
Tringa nebularia	1	132.00	1.05	0	0	56º12'N	16º24'E	200.85	17.80	15.30	20.60	3.95	[4]	
Tringa ochropus	2	68.00	0.62	0	0	56º12'N	16º24'E	200.85	17.80	15.30	20.60	3.95	[4]	
Tringa glareola	29	53.00	0.53	0	0	56º12'N	16º24'E	200.85	17.80	15.30	20.60	3.95	[4]	
Actitis hypoleucos*	3	46.55	0.47	1	1	39º01'N	5º58'W	132.09	9.60	5.33	16.10	3.99	[3]	
Actitis hypoleucos	9	38.00	0.37	0	0	56º12'N	16º24'E	200.85	17.80	15.30	20.60	3.95	[4]	
Arenaria interpres*	5	100.00	0.56	0	1	11º40'N	15º45'W	265.44	31.07	23.37	41.03	3.52	[1]	
Arenaria interpres	1	94.00	0.99	0	0	76º25'N	87º06'W	120.84	0.53	-1.15	2.06	3.30	[8] 	
Arenaria interpres	2	87.00	0.92	0	0	76º25'N	112º15E	140.84	1.97	0.23	3.67	4.19	[9]	
Arenaria interpres	2	88.00	0.74	0	0	56º12'N	16º24'E	200.85	17.80	15.30	20.60	3.95	[4]	
Arenaria interpres*	3	114.00	0.99	0	1	53º11'N	6º36'E	125.01	15.00	5.00	10.00	2.00	[2]	
Calidris tenuirostris	5	198.20	1.08	0	0	30º48'N	121º27'E	183.76	15.40	10.20	21.30	4.26	[10]	
Calidris tenuirostris	5	122.20	1.85	0	0	18º00'S	122º22E	255.02	30.55	24.05	40.95	4.53	[10]	
Calidris canutus*	13	115.00	0.83	0	1	11º40'N	15º45'W	265.44	31.07	23.37	41.03	3.52	[1]	
Calidris canutus	3	97.00	0.67	0	0	56º12'N	16º24'E	200.85	17.80	15.30	20.60	3.95	[4]	
Calidris canutus*	13	125.00	0.90	0	1	11º30'N	15º55'W	256.69	30.60	22.85	42.50	3.51	[11]	
Calidris canutus*	8	142.00	1.02	0	1	53º29'N	06º12'E	34.17	8.00	6.66	9.40	7.48	[11]	
Calidris canutus*	13	119.00	0.84	0	1	33º05S	18º02E	243.35	22.80	16.37	33.00	4.88	[1]	
Calidris alba*	4	47.00	0.55	0	1	11º40'N	15º45'W	265.44	31.07	23.37	41.03	3.52	[1]	
Calidris alba	2	46.50	0.55	0	0	67º12'N	41º17'E	142.51	8.15	8.13	8.19	4.61	[9]	
Calidris alba*	7	48.00	0.51	0	1	33º05S	18º02E	243.35	22.80	16.37	33.00	4.88	[1]	
Calidris alba	1	47.00	0.50	0	0	76º25'N	112º15E	140.84	1.97	0.23	3.67	4.19	[9]	
Calidris minuta	3	22.33	0.36	0	0	72º24'N	139º32'E	149.18	4.05	1.97	6.23	5.75	[9]	
Calidris minuta	3	30.36	0.31	1	1	39º01'N	5º58'W	132.09	9.60	5.33	16.10	3.99	[3]	
Calidris minuta	1	20.00	0.33	0	0	56º12'N	16º24'E	200.85	17.80	15.30	20.60	3.95	[4]	
Calidris minuta	1	22.00	0.33	0	0	70º11'N	67º17'E	147.51	8.26	4.17	12.60	4.38	[9]	
Calidris minuta	1	23.00	0.42	0	0	67º12'N	41º17'E	142.51	8.15	8.13	8.19	4.61	[9]	
Calidris minuta	2	24.00	0.39	0	0	68º53'N	53º34'E	144.59	8.92	6.52	11.30	4.26	[9]	
Calidris ferruginea*	5	57.00	0.56	0	1	11º40'N	15º45'W	265.44	31.07	23.37	41.03	3.52	[1]	
Calidris ferruginea	2	51.50	0.57	0	0	70º11'N	67º17'E	147.51	8.26	4.17	12.60	4.38	[9]	
Calidris ferruginea	6	49.00	0.46	0	0	56º12'N	16º24'E	200.85	17.80	15.30	20.60	3.95	[4]	
Calidris ferruginea*	10	50.00	0.54	0	1	33º05S	18º02E	243.35	22.80	16.37	33.00	4.88	[1]	
Calidris ferruginea	1	47.00	0.60	0	0	72º24'N	139º32'E	149.18	4.05	1.97	6.23	5.75	[9]	
Calidris ferruginea	1	50.00	0.72	0	0	76º25'N	112º15E	140.84	1.97	0.23	3.67	4.19	[9]	
Calidris ferruginea	2	53.00	0.63	0	0	73º05'N	70º07'E	134.59	1.68	2.64	6.11	5.43	[9]	
Calidris maritima	4	58.00	0.71	0	0	76º25'N	87º06'W	120.84	0.53	-1.15	2.06	3.30	[8]	
Calidris alpina*	1	35.00	0.35	0	1	11º40'N	15º45'W	265.44	31.07	23.37	41.03	3.52	[1]	
Calidris alpina	1	40.00	0.47	0	0	67º12'N	41º17'E	142.51	8.15	8.13	8.19	4.61	[9]	
Calidris alpina*	41	45.79	0.44	0	1	39º01'N	5º58'W	132.09	9.60	5.33	16.10	3.99	[3]	
Calidris alpina	30	41.00	0.46	0	0	56º12'N	16º24'E	200.85	17.80	15.30	20.60	3.95	[4]	
Calidris alpina	1	43.00	0.55	0	0	70º11'N	67º17'E	147.51	8.26	4.17	12.60	4.38	[9]	
Calidris alpina	10	48.22	0.60	0	1	36º23'N	6º8'W	218.77	16.20	13.10	20.50	3.80	[3]	
Calidris alpina	4	45.50	0.55	0	0	73º05'N	70º07'E	134.59	1.68	2.64	6.11	5.43	[9]	
Calidris bairdii	3	35.10	0.43	0	0	70º34'N	128º16'W	158.76	5.47	2.62	8.26	3.90	[8]	
Calidris bairdii	2	34.20	0.43	0	0	74º33'N	82º50W	124.18	0.04	-2.91	2.77	3.86	[8]	
Limicola falcinellus	1	35.00	0.43	0	0	56º12'N	16º24'E	200.85	17.80	15.30	20.60	3.95	[4]	
Calidris fuscicollis	4	34.88	0.47	0	0	70º34'N	128º16'W	158.76	5.47	2.62	8.26	3.90	[8]	
Calidris fuscicollis	1	33.20	0.47	0	0	76º25'N	87º06'W	120.84	0.53	-1.15	2.06	3.30	[8]	
Calidris fuscicollis	10	34.91	0.51	0	0	74º33'N	82º50W	124.18	0.04	-2.91	2.77	3.86	[8]	
Calidris fuscicollis	3	34.00	0.42	0	0	68º25'N	66'56'W	150.01	2.46	0.49	4.42	3.91	[8]	
Calidris minutilla	1	18.50	0.34	0	0	70º34'N	128º16'W	158.76	5.47	2.62	8.26	3.90	[8]	
Calidris pusilla	5	20.00	0.31	0	0	69º32'N	139º33'W	150.01	6.05	3.53	8.66	4.74	[8]	
Calidris melanotos	2	65.00	0.69	0	0	69º32'N	139º33'W	150.01	6.05	3.53	8.66	4.74	[8]	
Calidris himantopus	1	48.00	0.64	0	0	69º32'N	139º33'W	150.01	6.05	3.53	8.66	4.74	[8]	
Philomachus pugnax (f)*	12	107.30	0.82	1	1	39º01'N	5º58'W	132.09	9.60	5.33	16.10	3.99	[3]	
Philomachus pugnax (f)	2	87.00	0.75	0	0	56º12'N	16º24'E	200.85	17.80	15.30	20.60	3.95	[4]	
Philomachus pugnax (m)*	10	177.89	1.35	0	1	39º01'N	5º58'W	132.09	9.60	5.33	16.10	3.99	[3]	
Philomachus pugnax (m)	6	136.00	1.10	0	0	56º12'N	16º24'E	200.85	17.80	15.30	20.60	3.95	[4]	
Phalaropus lobatus	1	27.00	0.36	0	0	56º12'N	16º24'E	200.85	17.80	15.30	20.60	3.95	[4]	
Phalaropus fulicaria	4	40.53	0.43	0	0	73º39'N	115º39'W	148.35	2.34	-0.55	5.08	5.17	[8]	
Tryngites subruficollis	4	50.50	0.61	0	0	73º39'N	115º39'W	148.35	2.34	-0.55	5.08	5.17	[8]	
Note: m, males; f, female	
                                                                                                                                                                                                                                                                                          

References for Appendix S1
1. 	Kersten M, Bruinzeel L, Wiersma P, Piersma T (1998) Reduced basal metabolic rate of migratory waders wintering in coastal Africa. Ardea 86: 71–80.
2. 	Kersten M, Piersma T (1987) High levels of energy expenditure in shorebirds: metabolic adaptations to an energetically expensive way of life. Ardea 75: 175–187.
3. 	This study
4. 	Kvist A, Lindström Å (2001) Basal metabolic rate in migratory waders: intra-individual, intraspecific, interspecific and seasonal variation. Functional Ecology 15: 465–473.
5. 	Mathiu, PM, Johnson OW, Johnson PM, Whittow GC (1989) Basal metabolic rate of Pacific Golden-Plovers. The Wilson Bulletin 101: 652–654.
6. 	Duriez O, Pastout-Lucchini L, Boos M, Chastel O, Fritz H, et al. (2004) Low levels of energy expenditure in a nocturnal, forest-dwelling wader, the Eurasian Woodcock Scolopax rusticola. Ardea 92: 31–42.
7. 	Speakman JR (1984) The energetics of foraging in wading birds. PhD thesis, University of Stirling, Stirling, U.K.
8. 	Lindström Å, Klaassen M (2003) High basal metabolic rates of shorebirds while in the Arctic: a circumpolar view. Condor 105: 420. 
9. 	Lindström Å (1997) Basal metabolic rates of migrating waders in the Eurasian Arctic. Journal of Avian Biology 28: 87–92. 
10. 	Battley PF, Dekinga A, Dietz MW, Piersma T (2001) Basal metabolic rate declines during long-distance migratory flight in Great Knots. Condor 103: 838–844.
11. 	Piersma T, Bruinzeel L, Drent R, Kersten M, Meer J v d, et al. (1996) Variability in basal metabolic rate of a long-distance migrant shorebird (red Knot, Calidris canutus) reflects shifts in organ sizes. Physiological Zoology 69: 191–217. 
