# Supplementary material for: Avian BMR in Marine and Non-Marine Habitats: A Test Using Shorebirds
Source: PLoS One. 2012 Jul 31;7(7):e42206. doi: 10.1371/journal.pone.0042206 (PMC3409136; doi:10.1371/journal.pone.0042206)
Supplement: Appendix S2 — Phylogeny for the A 39 species (92 tips) of shorebirds included in the “full dataset” and for the B 25 species (39 tips) included in the “wintering dataset”. Both trees were derived from the shorebird supertree developed by Thomas et al. [1]. Branch lengths specified by Pagel’s [2] arbitrary method. (RTF) [file pone.0042206.s002.rtf]

Appendix S2. Phylogeny for the A 39 species (92 tips) of shorebirds included in the “full dataset” and for the B 25 species (39 tips) included in the “wintering dataset”. Both trees were derived from the shorebird supertree developed by Thomas et al. [1]. Branch lengths specified by Pagel's [2] arbitrary method.
A


B

References for Appendix S2

1.	Pagel MD (1992) A method for the analysis of comparative data. Journal of Theoretical Biology 156, 431–442.
2.	Thomas GH, Wills MA, Székely T (2004) A supertree approach to shorebird phylogeny. BMC Evolutionary Biology 4, 28.
